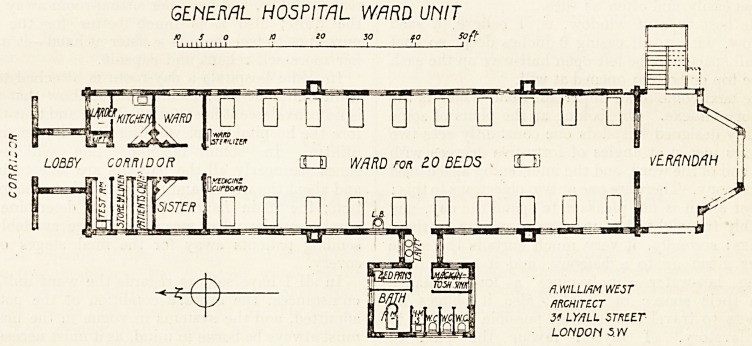# A Complete Ward Unit for a Modern General Hospital

**Published:** 1911-05-20

**Authors:** A. William West


### Hospital Architecture and Construction.

[Communications on this subject should be marked "Architecture" in the left-hand top corner of the envelope.]

### A COMPLETE WARD UNIT FOR A MODERN GENERAL HOSPITAL.

By A. WILLIAM WEST.

By a ward unit, I understand a ward for sick beds, and the necessary adjuncts for the treatment and convenience of the sick patient. This may be subdivided into medical and surgical, the surgical unit requiring an operation theatre, anæsthetising room, and sterilising room, either in close connection with it, or easily accessible, either for one unit or in common with two or three wards, and a splint room.

Then, again, the ward unit for special and separation cases is somewhat different in its requirements. In designing a modern hospital ward unit, there are many things to be taken into account, the first and foremost of which is the system of dividing the beds among the staff—that is, whether all the beds in a ward are in charge of one physician or surgeon, or whether they are allotted among two or more.

In the latter case it is obviously impossible to arrange quarters for the house officer adjoining his own ward.

I would summarise the requirements as follows: Floor space and cubic space per bed; a separate ward for one or two cases; lavatory accommodation; space for soiled linen and bed-pans and urines; ward kitchen and larder; sisters' room; scrubbers' room; patients' clothing; linen; test room; balcony.

The floor space may be generally taken to vary from 120 feet to 150 square feet per bed, the hospitals having medical schools requiring the larger space. The width of the wards is generally about 28 or 30 feet, and the height 13 feet. This is now considered to be the proper height for a ward, as it is contended, and I think rightly, that space above that is for all practical purposes thrown away, and therefore only an additional expense without any compensating advantage; thus a ward having 1,600 cubic feet per bed, would require to be 28 feet wide, 13 feet high, and the beds 9 feet from centre to centre. All angles should be rounded, and if electric light casings must project, they should be specially made, so as to harbour no dust; one electric light bracket should be placed over each bed, with a plug for movable light; besides these there should be two or three pendant lights down the centre of the wards in convenient positions for the night nurses' use, with green shades that can be raised or lowered at will, and one bracket with a separate switch to light up the medicine cupboard.

The heating of a ward is a difficult problem; it is not now considered necessary to keep up a high temperature, and as the benefits derived from Consumption sanatoria are being widely known, and people generally live with their windows open day and night more and more, the warming required will be quite easily obtained by open fire-places or stoves, without any heating apparatus in addition. At present, every precaution is taken to round angles, and yet in many cases, horrible pipes are trailed along the floors, to make inaccessible places and harbour dirt; or channels are sunk into the floor, which are never cleaned out. It is not the patient in bed that is cold, but the nurse, or those who go into the ward, and they can be suitably clothed. This is one of the great points upon which there is serious need of co-operation between medical men, hospital managers, and architects, in cases where enormous expense has been incurred in putting in heating apparatus of various kinds; how often is it really wanted? Nurses in their lectures, no doubt, are told that wards should be kept up to 60 degrees; the heat is automatically turned on or off by an engineer at certain seasons of the year, and there is an end of the matter.

As to ventilation, this is also passing away as a difficult problem with the advent of more fresh air and open windows. When one reads of the elaborate

GENERAL HOSPITAL WARD UNIT

schemes in use to draw off the used-up air along a series of shafts, generally brought into contact with hot-water cisterns or something of that kind to quicken the draught, and then, after dodging chimney stacks and any other obstructions there may be, being carried to the open air, one sometimes wonders if the authors of these devices ever heard of down-draughts, and stopped to think of the accumulated dirt in these shafts that can never be cleaned out. I believe open windows, and gratings with mica flaps opening into flues carried up with smoke flues, are all that are necessary, providing they are sufficiently large.

For the floors there is nothing to equal good teak laid on a fireproof base, the boards being 6-inch width and laid lengthwise with the ward, and polished. For the walls, many advocate a tile dado about 6 feet high and painted walls above, but, speaking personally, I much prefer a painted wall; the tile-work, whatever colour it may be, always gives a feeling of coldness and the impression of a larder, and a good enamel paintwork can be washed just as easily and often as tiles.

The best type of window, is, I believe, a sash window, with a sill casing 6 inches deep, so that a small space may be left open half-way up the sash or the top or bottom opened at will.

We next come to an important item, which is the lavatory annexe. On looking at the plans of some recently designed hospitals one constantly sees two annexes placed at angles of forty-five degrees with each end of the ward, and the intervening space used as a balcony. There are one or two objections to this: first of all, it is not pleasant to spend the day, and possibly the night also, in close proximity to these fittings; secondly, it very much curtails the space better given up to a balcony, and it necessitates taking hot-water supply-pipes a long distance from their source of supply; also it obliges all patients to travel the furthest possible distance to the lavatory. I myself favour the plan of placing a disconnecting lobby half-way down the ward, and from that to reach an annexe with w.c.s and bath, and bed-pan room, mackintosh sink, etc.; on one side of the lobby may be placed lavatory basins, and on the other side tanks for soiled linen, with a ventilated shoot leading to the ground or basement; the cross-ventilation of the wards is not interfered with by this method to any extent worth considering.

The doors to the w.c.s, etc., should be of teak and made to swing, so that there should be no noise from the banging of doors. The partitions between the apparatus should be kept six inches from the floor, to enable a thorough cleansing, and left open above, eight feet from the ground, so as to obtain plenty of ventilation. In each ward there should be placed in front of a window a sink with hot and cold water for the use of the staff, as it saves time in the emptying and filling of basins.

I believe a balcony at least eight feet wide should be placed at the end of the ward, the full width of the ward, from which an outside staircase can lead to the ground, which is useful for ordinary purposes, or in case of fire; a gate, if necessary, can be placed leading on to the stairs, which can be locked with a glass bolt, so as to be breakable in case of fire.

This arrangement I believe to be infinitely preferable to a balcony placed the whole length of the ward, which must interfere with both light and ventilation and is not easy to roof in, whereas a little elongation of the ward roof easily covers the balcony at the end of the ward. Also, there should be a small ward for one or two beds for noisy or suspicious cases while under observation; in addition to this, there should be a ward kitchen, with a sink, a ward-maid's room for brooms, brushes, etc., and a test-room, and in the case of a surgical ward a room for splints, and in connection with the kitchen a lift from the main kitchen, to supply diets; also a sisters' sitting-room, although I am inclined to think this will soon be recorded among the things no longer wanted, as the idea is gaining ground rapidly that while a sister is on duty she would do all her work in the ward, where a writing-table can easily be provided, so that when she is off duty she can be really off duty, without any feeling of responsibility, and have her sitting-room away from the ward; also it is much better for the staff nurse not to feel there is a sister at hand—it makes her more self-reliant and capable.

In some hospitals a day-room is attached to the ward unit, but this, I fancy, must show that there is no convalescent hospital available, and that therefore the hospital is not working up to its maximum utility. In some hospitals one will sometimes see nearly all the patients are able to be up and about the wards, and in others this is the exception; the main factors that must determine this point must be what means there are available for sending patients away for the final stages of recovery.

In all I have said as regards the ward unit, circumstances, the average condition of the patients admitted, and the systems in vogue in the hospital must always be borne in mind, as it must necessarily affect the accommodation required and its disposition, and therefore no hard and fast rules can be laid down.

That there has been enormous improvement in the ward unit of to-day cannot be questioned; as there has been in the past so doubtless there will be in the future, the architect working side by side with the medical staff and nurses, quick to accommodate himself to new conditions. That he has proved himself capable of this will, I think, be realised when one reads of the condition of things in the past. Reading the other day an account of the old St. Thomas' Hospital, I came across the following in reference to the wards: "They are generally well ventilated, kept at a uniform and agreeable temperature by two fires, and in cold weather by hot-water apparatus, and are generally quite free from offensive smells."

I wonder what would be said of the architect of the present day who designed any part of a hospital in which all that could be said was that it was generally quite free from offensive smells; and one wonders what the condition of the wards must have been elsewhere at this time.

I think I have now mentioned the chief structural requirements of a ward unit, and will proceed to the chief accessories in the way of furniture.

The chief item, of course, is the bedstead. This should be of iron, enamelled. And beware of the white enamel bed; when new it certainly looks very nice, but the least mark shows, and in a very short space of time it looks shabby, and no amount of patching ever makes it look nice, so it is best to have dark blue or black. It should have a wire mattress, of which there are several good patterns on the market (stretcher boards can be placed for fracture cases). It will be found convenient to have two large castors at the head, and knobs at the foot, as then the bed does not move during a dressing, but can very easily be moved by slightly raising the foot end if required. It is well to remember not to have rubber pads on the knobs or castors, as polished floors very soon make them perish; brass castors and wood knobs will be found best. The bedsteads as made now can be supplied with an attachment for a pulley if required, and also for a very simple arrangement for a tent when wanted. The bedsteads should all be of a uniform size, or much inconvenience will be caused in getting the right mattresses; and finally the bedsteads should be of such a height as to minimise as far as possible the necessity for stooping on the part of the nurses when attending to a patient.

Against the wall, either to right or left of the door upon entering the ward, it will be found convenient to have a lock-up medicine cupboard with a light over it, in a convenient position for seeing the contents at night: that no mistakes may be made, a section of it, under a separate key, should be kept for poisons. Upon the opposite side may be placed an intercommunicating telephone and a fire-alarm in a glass case.

A steriliser for ward instruments should be placed in a convenient position with a gas-jet, so that it can always be kept in use.

A fitting may be placed centrally in the ward with drawers and cupboards for ward stores, the top of which acts as a table, and it is a good plan to have this of teak with a movable glass top, as it prevents the polish being injured and can be insured for a small sum.

A wheel chair or two should be provided for patients able to wheel themselves about and a few light screens covered with washable material.

Then, finally, a locker for each bed. The most convenient form of this I know is a locker which forms a seat, with a back part with a shelf upon which can be placed a bottle or glass, and under which can be hung a towel, with a flap which can be held out by brackets to form a table over the bed, the seat portion being made to open on top and in front and behind, with rounded angles so as to be easily cleaned; and one or two movable tables for dressings, etc.

## Figures and Tables

**Figure f1:**